# Promising approach for targeting ROBO1 with CAR NK cells to combat ovarian cancer primary tumor cells and organoids

**DOI:** 10.2144/fsoa-2023-0135

**Published:** 2024-07-29

**Authors:** Yan Zhu, Luanhong Wang, Biyu Jiang, Yini Wang, Qing Wu, Sihua Hong, Xiaojing Wang, Yuancheng Li, Tian Guan, Haoyu Zeng, Congzhu Li

**Affiliations:** 1 Department of Gynecological Oncology, Cancer Hospital of Shantou University Medical College, Shantou, Guangdong, China; 2 Department of Cancer Research, Guangdong Procapzoom Biosciences Co., Guangzhou, Guangdong, China

**Keywords:** CAR-NK, organoid, ovarian cancer, PBMCs, ROBO1

## Abstract

**Aim:** This study aimed to explore using peripheral blood mononuclear cell (PBMC)-derived chimeric antigen receptor (CAR) NK cells targeting ROBO1 as a personalized medicine approach for ovarian cancer. **Methods:** A two-step strategy generated ROBO1-targeted CAR NK cells from PBMCs of ovarian cancer patients. Efficacy was evaluated using xCELLigence RTCA, CCK-8 and Live/Dead fluorescence assays. **Results:** ROBO1-NK cells exhibited higher efficiency in eradicating primary ovarian cancer cells and lysing ovarian tumor organoids compared with primary NK cells without ROBO1-CAR modification. **Conclusion:** These findings highlight the potential of developing ROBO1-targeted CAR-NK cells from patients' PBMCs as a personalized treatment option for ovarian cancer.

## Plain language summary

Ovarian cancer represents a formidable clinical challenge necessitating the urgent exploration of novel therapeutic approaches. In this study, the focus was directed toward ROBO1, a molecule known to play a pivotal role in cancer angiogenesis and metastasis, while limited investigation in the context of ovarian cancer. Leveraging this knowledge, we sought to construct ROBO1-targeting chimeric antigen receptor natural killer (CAR-NK) cells utilizing peripheral blood mononuclear cells derived from the patients themselves. The overarching goal of this investigation was to harness the potential of immunotherapy using autologous resources to realize personalized treatment strategies for ovarian cancer in clinical settings.

## Tweetable abstract

This study described a novel, autolougs immunotherapy, PBMC-derived ROBO1-targeted CAR NK cells for ovarian cancer treatment.

## Abstract (plain language summary)

This study introduces a novel autologous immunotherapy approach for ovarian cancer treatment, utilizing PBMC-derived ROBO1-targeted CAR NK cells.

The successful construction of CAR-NK cells using patient-derived PBMCs eliminates the risks associated with heterologous infusion.

The investigation highlights the potential of ROBO1 as an immunotherapy target in ovarian cancer.

ROBO1-NK cells demonstrate remarkable efficacy in lysing primary cells and organoids derived from patient-specific ovarian tumor tissues.

## Graphical abstract

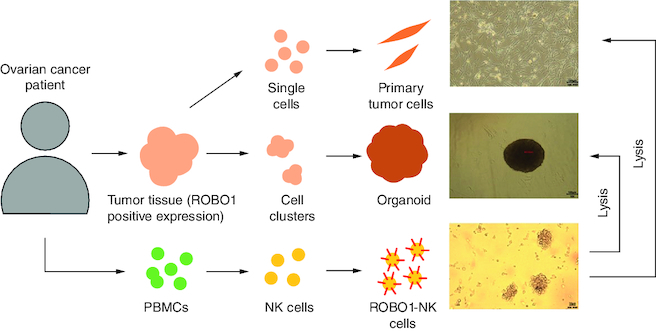

Ovarian cancer, a malignancy that affects the ovaries, has encountered limited success in its treatment regimens utilized in clinical settings [1]. This disease is a significant contributor to cancer-related mortality in women, ranking as the fifth leading cause of death. Annually, it affects approximately 240 thousand new patients, subjecting them to immense physical suffering [2,3]. Adding to the gravity of the situation, the 5-year survival rate for ovarian cancer patients stands at a mere 47% [4]. These statistics underscore the inadequacies of conventional chemotherapy approaches, emphasizing the pressing necessity for the identification of novel therapeutic targets or methodologies aimed at rescuing those afflicted by this aggressive form of cancer.

The ROBO1 protein, a member of the ROBO protein family, functions as a transmembrane receptor for SLIT proteins. It has been observed that ROBO1 is highly expressed in tumor cells, and the SLIT-ROBO signaling pathway plays a pivotal role in tumor angiogenesis and metastatic processes [5–9]. Studies have demonstrated that increased expression of ROBO1 has tumorigenic effects in ductal cancer, prostate cancer and lobular breast cancer [9,10]. Furthermore, ROBO1 expression has been detected in the SKOV-3 ovarian cancer cell line, indicating its involvement in malignant progression [11]. Nevertheless, the clinical significance of ROBO1 in ovarian cancer is inadequately explored. Moreover, the ligand of ROBO1, SLIT2, can also interact with ROBO4, which is normally involved in vascular stability regulation [12]. These interactions complicate the selection of appropriate drugs for clinical use. Considering these findings, it is imperative to develop more effective immunotherapy strategies targeting ROBO1 in clinical ovarian cancer.

In recent years, immunotherapy has emerged as a promising approach for cancer treatment, with techniques such as CAR-T cell therapies, monoclonal antibody drugs, and activating cytokines exhibiting potential anti-tumor effects. Notably, CAR-T cell therapies have achieved remarkable success in leukemia treatment, resulting in the complete remission of the disease in a young girl [13]. However, CAR-T cells have inherent limitations, including the risk of inducing cytokine release syndrome and neurotoxicity [14]. In comparison, CAR-NK cells offer several advantages over CAR-T cells. They present a lower risk of cytokine release syndrome, neurotoxicity, off-target tumor effects, and graft-versus-host disease (GVHD) following allogeneic infusion [14–17]. Additionally, unlike T cells that freely expand within the host, the lifespan of CAR-NK cells is restricted, providing an additional level of control over the quantity of donor cells within the host [15]. Currently, CAR-NK cells can be sourced from various origins, such as NK cell lines, peripheral blood mononuclear cells (PBMCs), umbilical cord blood (UCBs) and induced pluripotent stem cells (iPSCs). In our study, we utilized PBMCs derived from ovarian cancer patients themselves to generate CAR-NK cells, aiming to mitigate the uncertain risks associated with allogeneic transplantation in future clinical applications.

In this study, we conducted a collection of peripheral blood mononuclear cells (PBMCs) from ovarian cancer patients exhibiting positive ROBO1 expression in their tumor tissues. These PBMCs were then utilized to transduce NK cells with a ROBO1 chimeric antigen receptor (CAR), resulting in the generation of ROBO1-targeted CAR NK cells (ROBO1-NK). Concomitantly, tumor samples were obtained from the same ovarian cancer patients for the generation of primary tumor cells and tumor organoids. Subsequently, we treated these primary tumor cells and tumor organoids with ROBO1-NK and observed a high efficacy of ROBO1-NK in effectively eliminating these targets.

## Materials & methods

### Patients & tissues samples

The tumor primary cells and tumor organoids were generated from the ovarian tumor tissues of ovarian cancer patients. The NK cells were cultured from PBMCs of those patients. All the collection work were performed under full acknowledgments and agreements of the patients. All the study process was approved by the ethics committee and met the ethics principle of the Cancer Hospital of Shantou University Medical College.

### ROBO1-NK construction & generation

The purification and generation of NK cells from patients' PBMCs were conducted following established protocols [18–20]. In brief, PBMCs were isolated from the patient's peripheral blood using the MACSprep™ PBMC Isolation Kit (130-115-169, Miltenyi Biotec, Bergisch Gladbach, GER) employing the density gradient centrifugation method. The isolated PBMCs were then cultured in KBM-581 medium (88-581-CM, Corning, New York, US) at a density of 2 × 10^6^ cells/ml. Subsequently, the NK cells stimulation was performed following the datasheet of the AMMS^®^ NK Cell Culture Kit (AS01-1, Seafrom Biotechnology, Beijing, CN) and referenced the protocols in the previous studies [21,22]. On the first day of culturing, besides the regents used in the kits, additional low-dose of human IL-2 Recombinant Protein (PHC0026, Gibco, USA) and NK Inducing Regent (IR0103, iPSyte Biotec, Guangzhou, CN) were added into the medium, which was subsequently refreshed every 3 days until day 9. Afterward, the mature NK cells were collected and suspended for electroporation-based transfection with a plasmid containing the ROBO1-CAR sequence, following the procedures outlined in a previous study [23]. Cell suspension at a density of 2 × 10^7^ cells/ml was transfected with a plasmid concentration of 120 μg/ml. The transfection process involved two pulses, with the first pulse set at 1600 V for 20 ms, followed by the second pulse at 650 V for 100 ms. Subsequent to transfection, the NK cells were cultured for an additional 24 h and sorted using flow cytometry with the Anti-CAR antibody (0799-R271-F, Sinobiological, Beijing, CN). The resulting ROBO1-NK cells were then monitored using flow cytometry with anti-CD3, anti-CD34, anti-CD16, and anti-CD45 antibodies (ab16669, ab81289, ab183354, and ab40763, Abcam, Cambridge, UK).

### Generation of primary tumor cells & tumor organoids

The generation of ovarian tumor organoids and primary tumor cells followed the protocols described in a previous study [24]. In brief, fresh ovarian tumor tissue was first washed twice with ice-cold DPBS for cleaning. The tissue was then moistened with non-serum DMEM/F-12 (31330038, Gibco) and cut into small pieces. To facilitate digestion, 0.125% trypsin (15050065, Gibco) was added to the tissue pieces and incubated for 5 min at 37 °C. The digestion was subsequently halted by adding DMEM/F-12 with 10% serum (FSS500, Excell Bio, Taicang, China). The resulting suspension was transferred into a 15 ml tube and centrifuged at 1000 rpm for 5 min. The pellet was then suspended and centrifuged again to remove residual trypsin. Afterward, the pellet was divided into two parts. One part was seeded in a flask to generate ovarian primary tumor cells, while the other part was embedded in Matrigel (356231, Corning, USA) and seeded into a 24-well plate to generate ovarian tumor organoids. Both the primary tumor cells and tumor organoids were cultured in DMEM/F-12 medium supplemented with 10% serum.

### Cell lines & culture conditions

The SKOV-3 ovarian cell line was obtained from Procell Biotech Corporation (CL-0215, Wuhan, China) and overexpressed ROBO1 using the pEE6.4 plasmid (GM-1013PA02, Genomeditech, Shanghai, China). The cells were maintained in 5A basal medium (M4892, Merck, USA) supplemented with 10% serum and cultured at 37 °C in a CO_2_ incubator. Passaging of the cell line was carried out every 3 days.

### Immunohistochemistry

A portion of fresh tumor tissue was collected for immunohistochemistry detection before the generation of primary tumor cells and tumor organoids. This tumor tissue was fixed in 4% paraformaldehyde, dehydrated, and embedded in paraffin. Thin slices, approximately 5 μm thick, were then obtained. The slices were rehydrated and treated with 3% hydrogen peroxide before being blocked with 10% bovine serum albumin (BSA, SRE0096, Sigma, USA) for 1 h. Following that, the sections were incubated at 4 °C overnight with primary antibody against ROBO1 (ab7279, Abcam, Cambridge, UK). For the detection step, a secondary antibody, specifically anti-rabbit IgG modified with HRP (ab97051, Abcam), was used. Finally, color development was achieved using a DAB staining kit (D405772, Aladdin, Shanghai, China).

### Cell viability assay

Cell viability of ovarian primary tumor cells or cell lines was assessed using the Cell Counting Kit-8 (CCK-8, C0039, Beyotime, Shanghai, CN) according to the instructions provided in the data sheet. In brief, cells were suspended in the appropriate medium and seeded into a 96-well plate with a density of 1 × 10^4^ cells per well. The following day, the cells were treated with medium containing NK cells (Control), PBMC-derived NK cells (PBMC-NK), and PBMC-derived ROBO1-targeted CAR NK cells (ROBO1-NK) for 24 h. Subsequently, the medium containing NK cells and dead cells was replaced with fresh medium. The remaining live cells were then incubated with 10 μl CCK-8 solution per well at 37 °C for 2 h. The absorbance, indicating the optical density (OD) value, was measured at 450 nm using a Microplate Reader (PT-3502B, Potenov, Beijing, China).

### Non-invasive real time cell analysis

To assess the time curve of ROBO1-NK cells lysing SKOV-3 cells, xCELLigence RTCA instrument (Agilent, USA) was utilized following the procedure outlined in the data sheet. In the experiment, SKOV-3 cells were suspended and seeded into E-Plate 16 wells (5469830001, Agilent) with a density of 3 × 10^4^ cells per well. After allowing the SKOV-3 cells to settle and initiate adherent growth, they were treated with the medium alone as a control or with ROBO1-NK, PBMC-NK, and Mock-CAR-NK cells at a density of 1 × 10^5^ cells per well. Recording was performed at 15-min intervals over a period of 24 h. The cell index of each group was normalized to the control group.

### Statistical analysis

Statistical analysis was performed using Prism GraphPad (Version 8.0, GraphPad Software Inc, USA) with either one-way or two-way ANOVA or Student's *t*-test, as appropriate. Quantification data were normalized to the control group. In the cell viability assay, the cell survival rate was calculated using the formula:

$$\text{Cell survival rate}=\left[\frac{\left(\text{As}-\text{Ab}\right)}{\left(\text{Ac}-\text{Ab}\right)}\right]\times 100\%$$

were as represents the absorbance of the experimental well, Ac represents the absorbance of the control well, and Ab represents the absorbance of the blank well. For the quantification of organoid lysis, the lysis ratio was calculated as 100% – (FI × FA), where FI represents fluorescence intensity and FA represents fluorescence area normalized to the control group. p-values ≤0.05 were considered statistically significant.

## Results

**Figure 1.**
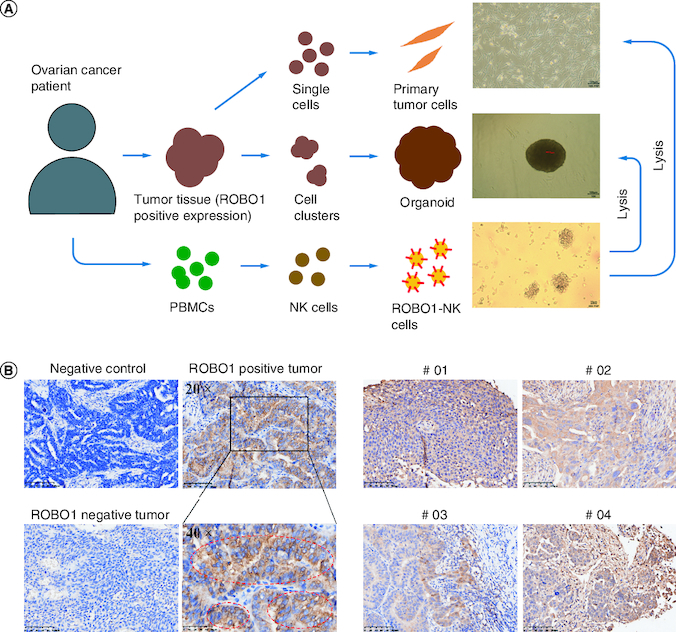
Strategy and materials in this study. **(A)** Diagram of the procedure in the study. The primary tumor cells and tumor organoids were generated from patients' tumor tissues and ROBO1-NK were constructed from patients' PBMCs. **(B)** IHC of ovarian tumor tissues. Negative control was used irrelevant IgG replacing primary antibody for incubating. Strong positive of ROBO1 expression on cell membrane was marked by red circles. **(C)** ROBO1 positively expressed in tumor tissues of the clinical ovarian cancer patient #01, #02, #03 and #04.

The experimental approach employed to generate ROBO1-NK cells and evaluate their anti-tumor efficacy against ROBO1-positive ovarian cancer cells is illustrated in Figure 1A. Initially, immunohistochemistry (IHC) analysis was performed to detect the presence of ROBO1 protein in ovarian cancer tumor tissues obtained from patients. Tissues showing positive ROBO1 expression were used to establish primary tumor cells and tumor organoids. Simultaneously, peripheral blood mononuclear cells (PBMCs) isolated from the respective patients were employed to generate ROBO1-NK cells. This innovative targeting approach aimed to investigate the potential for ovarian cancer treatment. Through IHC analysis, cytoplasmic and membrane expression of ROBO1 was observed in ovarian cancer cells, with some cells exhibiting robust positive ROBO1 expression surrounding the membrane (Figure 1B). In the clinical context, four out of fifteen ovarian cancer samples were identified as displaying positive ROBO1 expression (Figure 1C).

**Figure 2.**
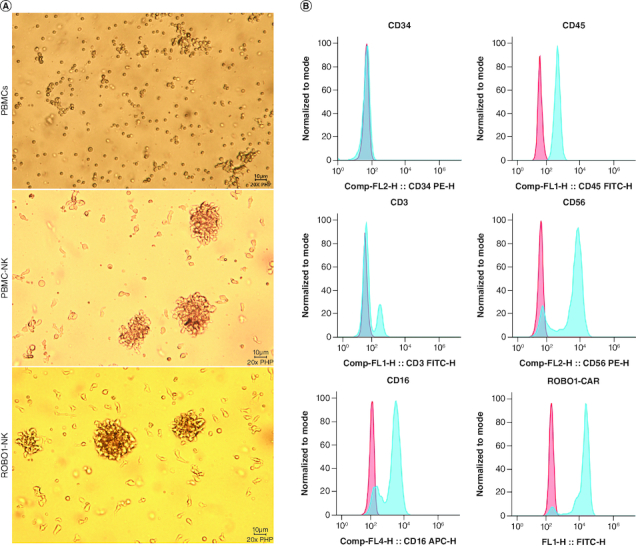
Characters of ROBO1-NK cells. **(A)** Morphology comparison of PBMCs (upper panel), PBMC-NK cells (mid panel) and ROBO1-NK cells (bottom panel) under bright field. **(B)** Biomarkers determination of ROBO1-NK cells by flow cytometry. For the biomarkers CD34, CD45, CD3, CD56 and CD16, negative controls were used irrelevant IgG replacing primary antibody for incubating, presented as red peaks. For the ROBO1-CAR detection, negative control was used PBMC-NK incubating with anti-CAR antibody, presented as red peak. Samples in detection were presented as blue peaks.

The detailed methods for the generation and construction of ROBO1-NK cells were described in the Materials and Methods section. Briefly, peripheral blood samples were collected from ovarian cancer patients, and peripheral blood mononuclear cells (PBMCs) were isolated (Figure 2A, upper panel). The NK cells were then stimulated to mature using interleukin-2 (IL-2) and subsequently harvested. The ROBO1-CAR sequence was introduced into the NK cells, followed by purification of the ROBO1-NK cells through flow cytometry separation. Comparisons with PBMC-NK cells (Figure 2A, middle panel) revealed that the ROBO1-NK cells did not exhibit cellular damage or morphological alterations (Figure 2A, lower panel). Protein expression analysis demonstrated that the majority of ROBO1-NK cells displayed the characteristic surface markers of peripheral blood NK cells, including CD34-, CD45^+^, CD3-, CD56^+^ and CD16^+^ (Figure 2B). Furthermore, positive expression of the ROBO1-CAR on the ROBO1-NK cells (Figure 2B) indicated that the successful transfer of the ROBO1-CAR and the purification processes using flow cytometry were effective in constructing the ROBO1-NK cells.

**Figure 3.**
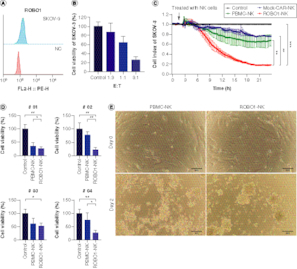
ROBO1-NK lyse ovarian cancer cell line and primary cancer cells. **(A)** Positive expression of ROBO1 on SKOV-3 cells. **(B)** Rate of SKOV-3 cell lysis with different E-T ratio. E: number of effective cells; T: number of target cells. **(C)** Time curve of different NK cells, including Mock-CAR-NK (blue), PBMC-NK (green), ROBO1-NK (red) and control (medium, black), lysing SKOV-3 cells. **(D)** Lysis rate of primary ovarian tumor cells from patient #01, #02, #03 and #04 by PBMC-NK and ROBO1-NK after 24 h, detected with CCK-8 assay. **(E)** Photograph of PBMC-NK and ROBO1-NK lysing efficacy on primary ovarian tumor cells after 48 h co-culturing. The primary cells were presented as spindle-like form while NK cells were spherical form with clusters.

In our *in vitro* investigations, we selected the SKOV-3 cell line as our experimental model, given its notable expression of ROBO1 (Figure 3A). When we treated SKOV-3 cells with varying ratios of ROBO1-NK cells, we observed substantial efficacy of the ROBO1-NK cells in inducing lysis of the SKOV-3 cells, particularly at the effector-to-target (E: T) ratios of 1:1 and 3:1 (Figure 3B). The measurement with the E:T ratios of 10:1 and 30:1 exhibit a complete lysis but not clear gradient trend in the co-culturing of SKOV-3 cells and ROBO1-NK cells (Supplementary Figure 2). To further investigate the cytotoxicity of ROBO1-NK cells, we monitored the xCELLigence RTCA data continuously over a 24-h period. The data revealed that NK cells derived from peripheral blood mononuclear cells (PBMCs) with a non-relevant chimeric antigen receptor (CAR) design (Mock-CAR-NK) or without the ROBO1-CAR (PBMC-NK) exhibited limited effectiveness, resulting in the lysis of SKOV-3 cells to only 76 and 67%, respectively, as evidenced by the decline in the cell index from 1.0 to 0.76 and 0.67. In contrast, ROBO1-NK cells demonstrated superior efficacy, leading to the lysis of SKOV-3 cells from 100 to 18% (Figure 3C). Based on these promising findings, we obtained four ROBO1-positive samples from ovarian cancer patients and derived primary tumor cells to evaluate the effectiveness of ROBO1-NK cells. Utilizing the CCK-8 assay, we observed that following 24 h of co-culturing, ROBO1-NK cells efficiently lysed the primary tumor cells from sample #01, #02, #03 and #04, resulting in lysis rates of 26, 23, 52 and 27%, respectively, while PBMC-NK cells exhibited lysis rates of 36, 78, 61 and 75% (Figure 3D). After 48 h, ROBO1-NK cells almost completely eradicated the spindle-type primary tumor cells, whereas PBMC-NK cells left some residual cells (Figure 3E).

**Figure 4.**
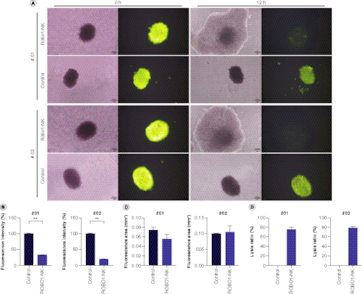
Detection of ROBO1-NK lysing ovarian cancer organoids. **(A)** Bright field and fluorescent images of organoids after treatment with ROBO1-NK cells or medium as control. Organoids presented as lager spherical form with green fluorescence, ROBO1-NK cells presented as smaller spherical form around the Organoid without fluorescence. The photographs were taken at the beginning of h 0 to the lysis finishing of h 12. **(B)** Quantification of fluorescence intensity of organoids, indicating the cells viability in organoids. **(C)** Quantification of fluorescence area of organoids. **(D)** Calculation of lysis ratio of organoid after treatment with ROBO1-NK cells.

To correspond with the translational focus of this investigation, we employed patient tumor organoids as our *ex vivo* model to extend our investigation into the lysing potential of ROBO1-NK cells. Ovarian cancer organoids derived from sample #01 and #02 were established, stained using Live/Dead cell fluorescence, and subsequently co-cultured with ROBO1-NK cells overnight. The comparison results of the IHC staining between tumor tissues and the organoids were detected with CA125, HE4 and CEA (Supplementary Figure 4) as the biomarker of the ovarian cancer [25,26]. From the results, we observed that both the ovarian cancer tissue and the organoid presented cytoplasmic positive expression of CA125 and HE4, and membrane positive of CEA. When co-culturing the organoids with ROBO1-NK cells together, the photographic data clearly demonstrated that both the #01 and #02 organoids were lysed by the ROBO1-NK cells after 12 h of treatment. This was demonstrated by the disruption of organoid structure and the attenuated green fluorescence, in contrast to the control group where the fluorescence remained stable (Figure 4A). The fluorescence intensity was quantified and shown in Figure 4B, while the measurement of the fluorescence area is displayed in Figure 4C. Notably, the rate of lysis mediated by ROBO1-NK cells was significantly higher compared with the control group, with a lysis rate of 75% and 79% observed for organoids derived from samples #01 and #02, respectively (Figure 4D).

## Discussion

Our primary objective was to identify a promising biomarker for ovarian cancer and to develop a targeted therapeutic approach specific to this biomarker. Despite the extensive research on the biomarker ROBO1 in various tumor types, its involvement in ovarian cancer pathogenesis remains inadequately explored. This knowledge gap served as a driving force for our study, prompting us to investigate the expression of ROBO1 in clinical ovarian cancer samples and to devise a safe and efficient treatment strategy for ovarian cancer patients exhibiting ROBO1 expression.

In order to accomplish this objective, we employed an immunotherapy strategy utilizing ROBO1-NK cells derived from autologous peripheral blood, thus circumventing the detrimental effects commonly associated with chemotherapy and mitigating the potential risks inherent in heterologous treatments. Through the utilization of immunohistochemistry (IHC) analyses, it was observed that not all patients diagnosed with ovarian cancer exhibited the expression of ROBO1 within their respective tumor tissues. This significant finding aligns seamlessly with our overarching aim of tailoring treatment modalities to the individual, and consequently imparts a suggestion that peripheral blood mononuclear cell (PBMC)-derived ROBO1-NK cells may stand as a more favorable therapeutic alternative for patients within the clinical framework. On the contrary, it is important to acknowledge the limitations inherent to PBMC-derived CAR-NK cells, which extend beyond the scope of our own research. Primarily, the variability in PBMCs among individual patients presents a significant challenge. The proportion of native NK cells within PBMCs differs, ranging from 5% to 20% [27–29] and varies in terms of proliferative potential. Consequently, it becomes challenging to establish a standardized criterion for selecting NK cells suitable for CAR transfection. Moreover, another obstacle we encountered was the lack of consensus regarding the most effective methodology for generating PBMC-derived CAR-NK cells. Various techniques have been employed to isolate and stimulate NK cells from PBMCs in different studies [29,30]. Despite these challenges, we successfully managed to construct ROBO1-NK cells from PBMCs using our current protocols and remain committed to further enhancing their efficacy in future investigations.

The primary procedures conducted in our study closely adhered to the protocols outlined in the datasheet of the MACSprep™ PBMC Isolation Kit, which was employed for the isolation of PBMCs. Furthermore, we followed the guidelines provided by the AMMS^®^ NK Cell Culture Kit for stimulating NK cell proliferation. However, it is important to note that we made certain modifications to the experimental setup by utilizing different manufacturers' reagents for specific purposes. For example, instead of using the medium included in the kits themselves, we employed KBM-581 medium from Corning for culturing PBMCs. This modification significantly enhanced the efficiency of NK cell maturation, resulting in improved outcomes. In order to evaluate the purity and quality of NK cells derived from peripheral blood mononuclear cells (PBMCs), we employed flow cytometry to examine the expression levels of several key biomarkers. Specifically, we assessed CD34, a biomarker predominantly expressed on hematopoietic stem cells, CD45 for all leukocytes, CD3 for T cells, and CD56 for NK cells. Through this comprehensive analysis, we were able to confirm the successful separation of primary NK cells from the PBMCs, as indicated by the absence of CD34 expression and the presence of CD45 expression. Furthermore, the absence of CD3 expression in conjunction with the positive expression of CD56 substantiated the high purity of the isolated primary NK cells.

When the NK cells were resuspended and added to the wells or flasks, they displayed a tendency to self-assemble into clustered formations during the subsequent culturing or lysis period. This phenomenon, as depicted in Supplementary Figure 3, is consistent with findings from previous studies [21]. The morphology of ROBO1-NK cells remained unaltered, indicating that transfecting ROBO1-CAR did not induce permanent damage to the PBMC-derived NK cells (Figure 2A). In addition to analyzing the purity, we also assessed the functional characteristics of the primary NK cells. Notably, a majority of primary NK cells exhibited positive expression of CD16, signifying intact lytic activity subsequent to isolation. Visual inspection of the constructed and purified ROBO1-NK cells confirmed prominent expression of the CAR on most NK cells, as illustrated in Figure 2B.

Furthermore, a comparative analysis of the cytolytic activity was performed among PBMC-NK, ROBO1-NK, and Mock-CAR-NK cells. Assessment of cell index monitoring exhibited a substantial improvement in the efficacy of PBMC-NK cells upon engineering with ROBO1-CAR, whereas no discernible alteration was observed with the irrelevant CAR construct, as elucidated in Figure 3C. PBMC NK or Mock-CAR NK cells exhibited a maximum lysis rate of approximately 40% (resulting in a reduction of SKOV-3 index from 100% to 67% and 76%). Conversely, ROBO1-CAR NK cells demonstrated a lysis rate exceeding 80% (reducing SKOV-3 index from 100% to 18%), indicating a twofold increase in lysis compared with the other two types of NK cells, attributable to the presence of ROBO1-CAR (Figure 3C). Additionally, it is crucial to acknowledge that ROBO1 represents a novel potential target identified in this study, warranting further investigation in future clinical studies.

It was found that ROBO1-NK cells exhibited similar cytotoxicity against tumor cells as normal NK cells. However, the introduction of ROBO1-CAR significantly enhanced the recognition and engagement of NK cells with tumor cells. Tumor cells employ two major strategies to evade NK cell recognition: secreting soluble ligands that block normal NK cell receptors, and downregulating their own activating ligands [31,32]. Consequently, normal NK cells struggle to effectively eliminate tumor cells due to difficulties in targeting them. In contrast, ROBO1-NK cells can still effectively target ovarian cancer cells by virtue of their ROBO1-CAR, which recognizes the ROBO1 protein expressed on the membrane of ovarian cancer cells. To validate the recognition capability of ROBO1-NK cells, a recombinant protein, ROBO1-scFv-Histag, was customized using ROBO1-CAR recognizing fragments. Incubating this recombinant protein with SKOV-3 cells, with or without ROBO1 expression, demonstrated successful binding of the recombinant protein to ROBO1-positive SKOV-3 cells. (Supplementary Figure 1).

To faithfully replicate the clinical conditions encountered in ovarian cancer treatments, we elected to employ clinical primary tumor cells and organoids as our *ex vivo* models, rather than utilizing animal models. In our examination of the clinical patient samples, it was observed that all four primary tumor cells expressing ROBO1 were effectively lysed by ROBO1-NK cells, as depicted in Figure 3D. Nevertheless, tumor cells from sample #03 displayed a slower rate of lysis in comparison to the other three samples, potentially attributable to a weaker positive expression of ROBO1, as indicated in Figure 1C. Across a 48-h timeframe, the ROBO1-NK cells successfully eradicated the primary tumor cells, as illustrated in Figure 3E. Due to the limited availability of patient tumor samples, organoids could only be generated from samples #01 and #02 due to their larger tumor volumes. Unfortunately, samples #03 and #04 yielded insufficient material for organoid generation. Nonetheless, our findings still underscore the potential of ROBO1-NK cells to effectively target tumor tissues at the organoid level, exemplified by our observations in the two ovarian cancer samples. To provide further clarity, the ovarian cancer organoids were subjected to Live/Dead cell fluorescence staining prior to their co-culture with NK cells. This staining allowed for the visualization of green fluorescence in the organoids, indicating their viability. As the organoids were subsequently lysed or killed by ROBO1-NK cells, the fluorescence was quenched, resulting in a loss of green fluorescence. The bright field images showcased a double-layered cell mass, depicting the situation where ROBO1-NK cells surrounded the organoids, with the organoids located at the core of the cell mass (Figure 4A). The results demonstrated that the organoids co-cultured with ROBO1-NK cells experienced rapid cell death, evident by the quenching of fluorescence. Conversely, organoids that were cultured alone remained viable and exhibited green fluorescence (Figure 4A). Surprisingly, the ROBO1-NK cells exhibited a substantially accelerated rate of lysing the ovarian cancer organoids compared with primary cancer cells in this investigation, achieving lysis within a mere 12 h (Figure 4A) in contrast to the 48 h needed for primary cancer cell lysis (Figure 3E). This striking disparity is likely attributable to various factors, including the concomitant viability of ROBO1-NK cells, the quantity of cells engaged in the treatment, and the intrinsic cellular composition of the organoids. Specifically, the proximity and concentration of ROBO1-NK cells surrounding the organoids appear to confer a more focused and efficacious lysing effect, as opposed to the dispersed distribution observed with primary cancer cells.

In conclusion, our study has unveiled a promising therapeutic strategy for ovarian cancer patients by targeting the novel biomarker, ROBO1. Through successful development and characterization, we have demonstrated the efficacy of ROBO1-NK cells derived from patients' own PBMCs in effectively lysing ovarian cancer cell lines, primary ovarian cancer cells, and tumor organoids. The clinical samples positive for ROBO1 reinforced the high anti-tumor potential of ROBO1-NK cells, highlighting their proficient targeting ability. We fully acknowledged the inherent heterogeneity of ROBO-1 expression within the samples of ovarian cancer patients. This diversity implies that ROBO-1 CAR-NK therapy may only be effective for a subset of patients. Furthermore, it is important to recognize that cancer itself is a complex ailment that necessitates more than a single drug for complete eradication. Our research aims to discover new strategies to overcome the challenges posed by cancer and to provide more feasible treatment options for a higher number of patients. Additionally, combining ROBO-1 CAR-NK therapy with other interventions shows promise as a potential approach. Our results inspire confidence in the future application of ROBO1-NK cells as a personalized and efficacious treatment approach for ovarian cancer patients.

## Conclusion

The present study elucidated a groundbreaking approach in the field of immunotherapy, wherein autologous PBMC-derived CAR NK cells targeting ROBO1 were employed for the treatment of ovarian cancer. The aim of the study was to evaluate the cytotoxicity of ROBO1-NK cells against pre-clinical models, patient-specific primary cells, and tumor-derived organoids from ovarian cancer patients. Remarkably, the findings of this investigation underscored the remarkable efficacy of ROBO1-NK cells in selectively eliminating tumor cells and organoids. These noteworthy results shed light on the potential of personalized therapeutic strategies for ovarian cancer, wherein CAR-NK cells can be engineered utilizing the PBMCs of individual patients.

## Supplementary Material

Supplementary Figures S1-S4

## Open access

This work is licensed under the Creative Commons Attribution 4.0 License. To view a copy of this license, visit http://creativecommons.org/licenses/by/4.0/
