# Supplementary material for: Promising approach for targeting ROBO1 with CAR NK cells to combat ovarian cancer primary tumor cells and organoids
Source: Future Sci OA. 2024 Jul 29;10(1):2340186. doi: 10.2144/fsoa-2023-0135 (PMC11290772; doi:10.2144/fsoa-2023-0135)
Supplement: Supplementary Figures S1-S4 [file IFSO_A_2340186_SM0001.docx]

**Supplementary materials**

**Figure S1** Determination of the ROBO1-scFv-Histag specific recognition. The ROBO1-scFv-Histag was incubated with SKOV-3 with or without ROBO1 expression (SKOV-3 or SKOV-3^ROBO1-/-^) simultaneously and analyzed by the flow cytometry. The SKOV-3 cell un-incubated with the scFv was used as the negative control.

**Figure S2**

The lysis efficiency of the ROBO1-NK cell at the E:T ratio of 1: 1, 3:1, 10: 1 and 30: 1 when co-cultured with the SKOV-3 cells.

**Figure S3**

Images of SKOV-3 cells at the day 0 before or after adding ROBO1-NK cells and the day 2 of co-culturing.

**Figure S4**

IHC staining of ovarian cancer tissues and the organoids with the antibodies of the ovarian cancer biomarker CA125, HE4 and CEA.

**Figure S1**


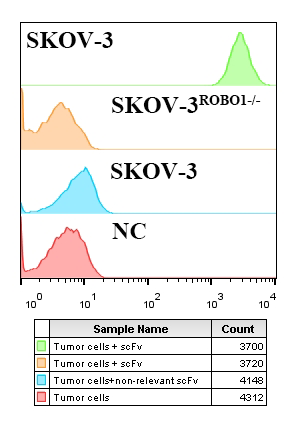


**Figure S2**


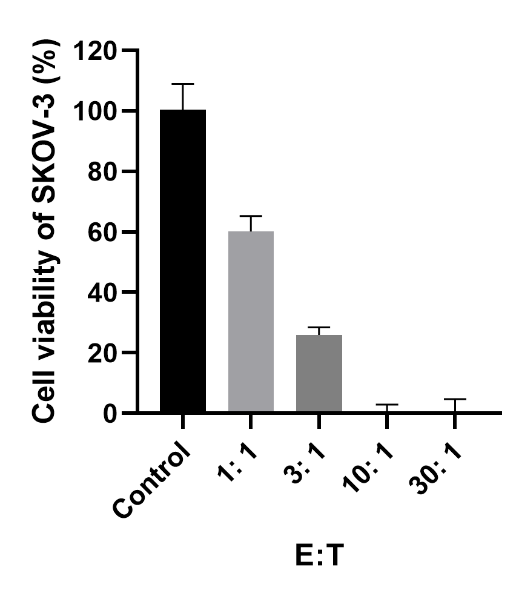


**Figure S3**


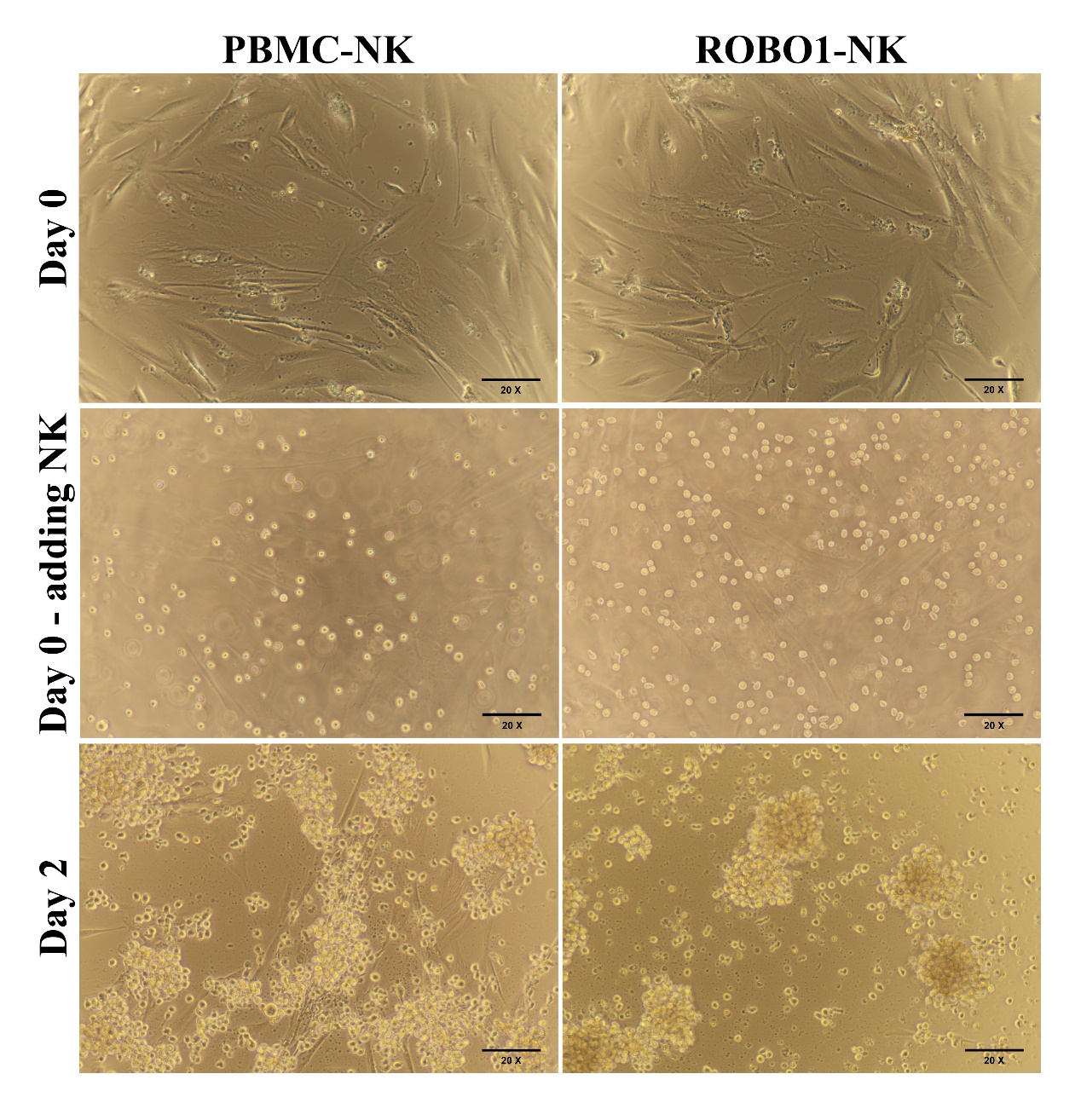


**Figure S4**

**
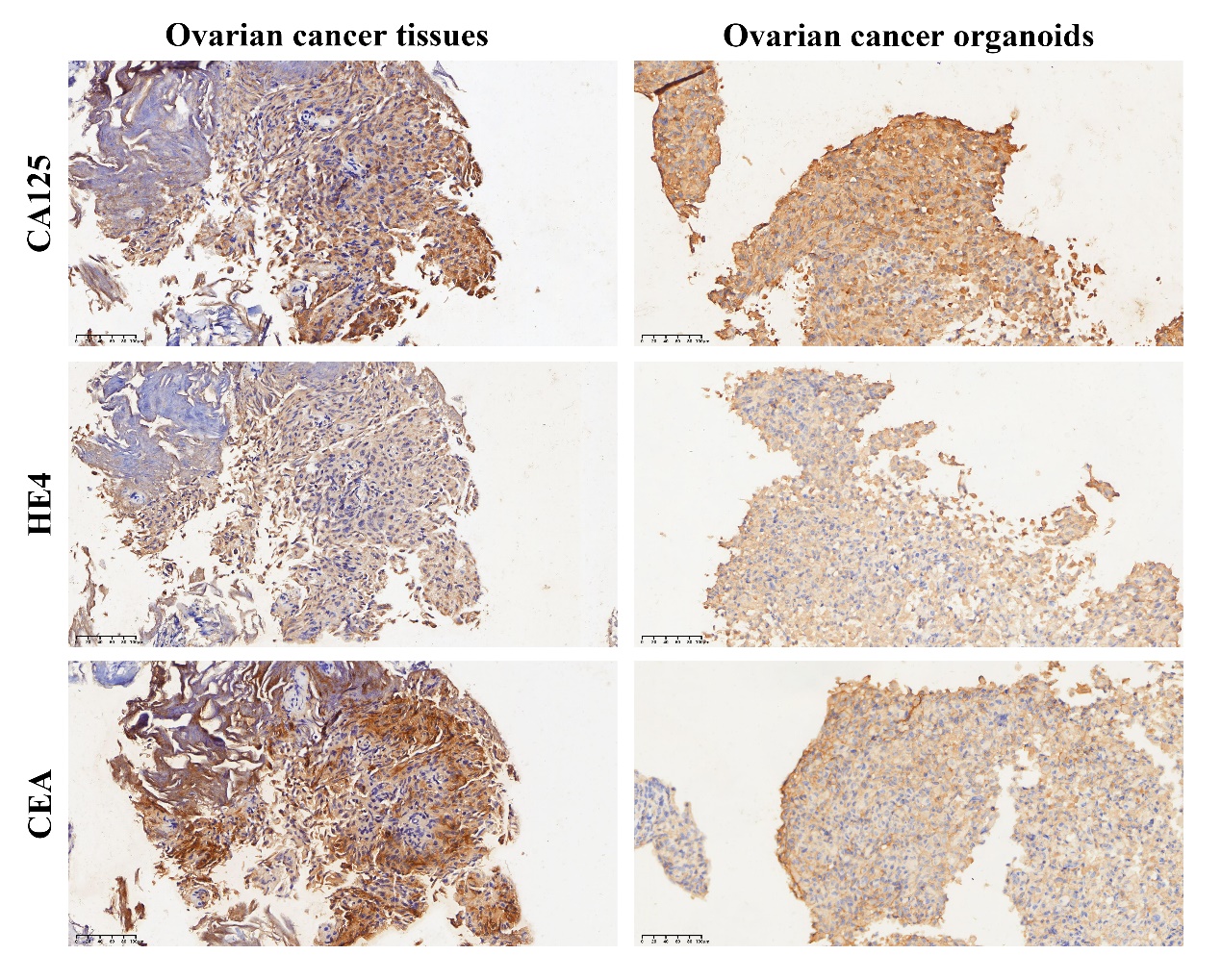
**
